# Upper back pain in postmenopausal women and associated physical characteristics

**DOI:** 10.1371/journal.pone.0220452

**Published:** 2019-07-31

**Authors:** Linda Spencer, Leanda McKenna, Robyn Fary, Angela Jacques, Kathy Briffa

**Affiliations:** School of Physiotherapy and Exercise Science, Curtin University, Perth, Western Australia, Australia; Ritsumeikan University, JAPAN

## Abstract

The physical characteristics of postmenopausal women that are associated with upper back pain are not well-understood. The aim of this cross-sectional study was to identify the physical characteristics associated with presence and severity of upper back pain in healthy postmenopausal women. Self-reported upper back pain presence (within the previous month) and severity (numerical rating scale) were examined against the physical characteristics: height; weight; body mass index; breast size; breast ptosis; upper back extensor muscle endurance (isometric chest raise test); head, shoulder and upper back posture (photogrammetry); thoracic extension mobility (photogrammetry); bone mineral density (dual-energy x-ray absorptiometry (DXA)); body composition (DXA); and thoracic kyphosis, thoracic osteoarthritis and thoracic vertebral fracture (all radiography). A multivariable logistic regression model, adjusted for age, was built using physical characteristics with a significant univariate association with upper back pain. Censored Tobit regression, adjusted for age, was used to examine each physical characteristic against upper back pain severity. Postmenopausal women (n = 119) with a mean (SD) age of 61.4 (7.0) years participated in the study. After adjusting for age, the physical characteristics independently associated with upper back pain were: height (OR: 0.50, 95% CI: 0.31–0.79); and upper back extensor muscle endurance (OR: 0.46, 95%CI: 0.28–0.75). This model explained 31% of the variance in upper back pain (*p*<0.001). After adjusting for age, being taller and having better upper back extensor muscle endurance were associated with lower odds for upper back pain. After adjusting for age, differences in upper back pain severity were explained by upper back extensor muscle endurance (*p* = <0.001) and lean mass (*p* = 0.01). Conclusion: As a modifiable physical characteristic of postmenopausal women with upper back pain, upper back extensor muscle endurance is worth considering clinically.

## Introduction

Upper back pain (UBP) describes pain in the region of the thoracic spine. It is a prevalent and disabling condition that contributes to a significantly reduced quality of life in women, particularly following menopause [1, 2]. Women are estimated to have a lifetime prevalence of UBP of between 6% [3] and 72% [4]. Risk factors related with UBP have not been clearly identified but could include an array of individual physical characteristics [4].

With a direct mechanical relationship to the upper back, physical characteristics such as poor posture [5]; spine mobility [6] and trunk strength [7, 8] have been associated with UBP in postmenopausal women. Similarly, decreased bone mineral density (BMD) [2, 9]; the presence of thoracic vertebral fractures [3]; and increasing thoracic kyphosis [2, 10, 11] have been related to an increased likelihood of UBP. However, these factors have not been simultaneously examined and interpretation of findings is complicated because many of these factors change with increasing age as well as being more likely following menopause.

Lack of clear definition of UBP has also hampered the evaluation of associated risk factors. Due to inadequate descriptions of the upper back region in prior epidemiological research, there is uncertainty as to which factors are related to the specific condition [4]. Whilst UBP could share a number of risk factors reported to be relevant to low back and neck pain, such as poor muscle function and non-neutral postures [12, 13], it is also likely that the condition has unique risk factors that relate more specifically to the anatomy and function of the upper torso and thoracic spine. A unique risk factor in women could be breast size, as this conceivably affects upper torso mechanics [14] and has been associated with UBP in postmenopausal women [15]. Breast size [16] and breast characteristics [17] commonly change following menopause, and whilst these characteristics have been reported to explain some of the variance in UBP in women aged 18–82 years (mean (SD) age 41 (19) years) [18], their importance and contribution to UBP alongside other potentially relevant physical characteristics, has not been previously explored.

Identifying factors that increase or decrease the risk of UBP in postmenopausal women could have significant clinical value. Specifically, knowledge of modifiable factors associated with changing the risk for UBP and its severity could be used to develop preventative and treatment strategies for the condition. Exploring these factors in postmenopausal women is particularly pertinent because they represent a growing proportion of general Australian population [19]. Since few studies have examined several physical characteristics collectively with well-defined UBP, neither the overall importance of these characteristics nor their importance relative to each other, is clear.

The aim of this study was to determine the physical characteristics of healthy postmenopausal women that were associated with the presence and severity of UBP. By exploring how women with UBP differ from those without UBP on the basis of several individual physical characteristics, we also aimed to highlight those most relevant in explaining the probability of UBP and those accounting for variability in the severity of UBP.

## Materials and methods

A cross-sectional study of postmenopausal women with and without UBP was conducted. Participants were excluded if they had menstruated in the past 12 months; reported a history of thoracic spine surgery, a systemic inflammatory condition, a neurodegenerative disorder, a known pathology of the breast, lung or thoracic spine; had cancer involving the bones; previous breast surgery; or long-term and recent ongoing use of steroid or pain medication.

Women were recruited via word-of-mouth, radio, newspaper and online advertising. The study was approved by the Human Research Ethics Committee at Curtin University. (Approval number RHDS-267-15) and all participants provided written informed consent.

A survey, accessible electronically via an emailed URL link (Qualtrics, version June 2016, Provo, Utah, USA) or in hard copy by post (and returnable in postage paid envelope) captured self-report information. Information included participants’ past medical history and UBP experienced within the past month.

Survey data were screened by a study supervisor (LM), who used consecutive sampling to sequentially invite at least 50 participants with UBP and 50 participants without UBP to attend a university-based health clinic on one occasion, for one-hour, to complete physical measures. Participants were asked to avoid unaccustomed activity that might induce soreness in the 48 hours prior to their allocated session. An experienced musculoskeletal physiotherapist (LS), blinded to the UBP status of participants, completed the physical measures in a standardised order on each participant. Following the session, participants attended a local radiological clinic at their convenience for a thoracic spine x-ray.

### Measured characteristics

Upper back pain was evaluated using the self-report information provided in completed surveys. Participants indicated the presence/absence of UBP within the previous month in response to a single question. The upper back was defined as the spine above the base of the rib cage and below the neck and a body diagram was provided for visual confirmation. Participants with UBP were asked to indicate the severity of their pain within the past month, using a numerical rating scale (NRS) of 0–10 (0 = no pain and 10 = worse pain imaginable) and the duration of their pain as acute (present for 0–3 months) or chronic (present for > 3 months).

Physical measures of participant’s height (m) and weight (kg) were used to calculate their body mass index (BMI) (kg/m^2^). Bone mineral density (BMD) (g/cm^2^), an averaged value from the left and right femoral neck of each participant, was measured using dual-energy x-ray absorptiometry (DXA) (GE Healthcare, Little Chalfont, UK). Lean mass (kg) and fat mass (kg) were measured using whole body DXA scans [20].

Breast size was calculated from a traditional measure of bra size [21]. Under-bust and over-bust measures that determined bra size [22] were converted into a continuous breast size score (BSS) (0–18) (see S1 Appendix) using a system conceptually similar to sizing unilateral breast prostheses [23]. Breast ptosis was the measured distance (cm) between the sternal notch and nipple with participants in sitting. Distances of the left and right sides were averaged. Breast splay was the measured distance (cm) between the left and right nipples whilst participants sat with their hands on their hips. Bra fit, that examined the band, cup, underwire, straps and front brand of the bra most frequently worn by a participant over the past month was categorized as ‘pass’ or ‘fail’ using professional criteria previously described by McGhee and Steele [24].

**Fig 1:**
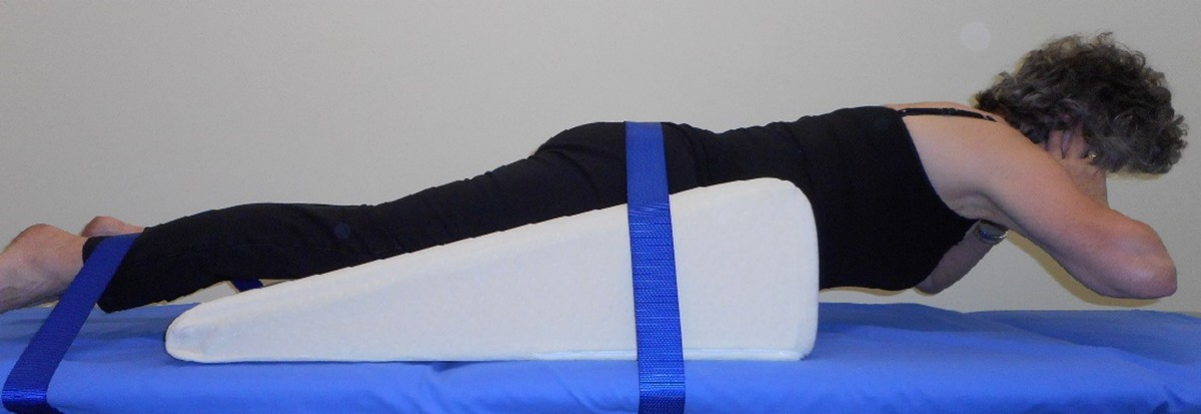
The isometric chest raise test.

Upper back extensor muscle endurance was tested using the isometric chest raise test [25]. This test was selected as it allowed assessment of endurance without confounding effects of poor extension mobility or a more kyphotic posture. The endurance of upper back extensor muscles was assessed in preference to the strength of the muscles in view of their primary function as postural muscles [26]. Participants were positioned in prone on a high density foam wedge cushion (Lunamumma, VIC, Australia) with the navel at the front edge of the cushion, arms unsupported and hands at the side of the head (Fig 1). The wedge cushion enabled participants with reduced extension mobility or large breasts to complete the test. Adjustable straps were used to fix the pelvis and lower limbs (below the knee) to the bed. Instructions to “lift your chest clear of the bed and maintain this position for as long as possible” were standardised. No encouragement was given to participants during the test. The time (s) to volitional fatigue, defined as the point at which the chest touched the bed, was recorded. Any participant who could not lift the chest from the bed to initiate the test would have been recorded as having a time of zero seconds. An upper limit cut-off time of 300s was imposed to terminate the test to avoid prolonged testing time. A hold time of 300s sufficiently surpasses the 75^th^ percentile for completion of the test by women over the age of 40-years according to normative data [8].

Upper back extension range of motion (back mobility) was determined using digital photography and methods analogous to those validated previously [27]. Photo-reflective markers were secured over the spinous processes of T1, T4, T8 and T12, identified by palpation downward from C7, before participants stood. Images (5152x3864 pixels) were taken from the left side at rest and in maximum extension. A digital camera (Nikon S3700, Nikon, Japan) on a tripod positioned 250cm away from participants, with the lens level with the mid-thoracic spine, was used. For the resting image, participants’ arms were elevated anteriorly to approximately 70° with hands supported on a 122cm pole. For the extension image, participants were instructed to fully elevate their arms, reach up and back as far as possible. Photographic images were analysed using ImageJ software (National Institutes of Health, Bethesda, MD) and thoracic angles were determined for each position as previously described [27]. The difference between measured angles represented the extension range of motion (°).

Thoracic kyphosis was measured using the vertebral centroid angle on a lateral radiograph [28]. The midpoints of four thoracic vertebral bodies (T1, T2, T11, T12) were used to calculate the angle of the thoracic curve in the sagittal plane as previously described by Harrison et al. [29]. Participants stood with their arms elevated to 90°. The x-ray device was positioned at a film focus distance of 120cm with the beam centered on the mid thoracic vertebrae. Centroid angles were determined digitally (InteleViewer, Inteleard, Montreal, Canada) by a single radiologist blinded to the clinical information of each participant. X-ray images were also used to assess osteoarthritis and vertebral fractures in each participant. Clinical judgement by the radiologist was used to define the osteoarthritis of the entire thoracic spine as either nil-mild or moderate-severe. Thoracic vertebral fractures were recorded for participants showing a ≥20% loss in vertebral body height with reference to normal adjacent vertebrae [30].

**Fig 2:**
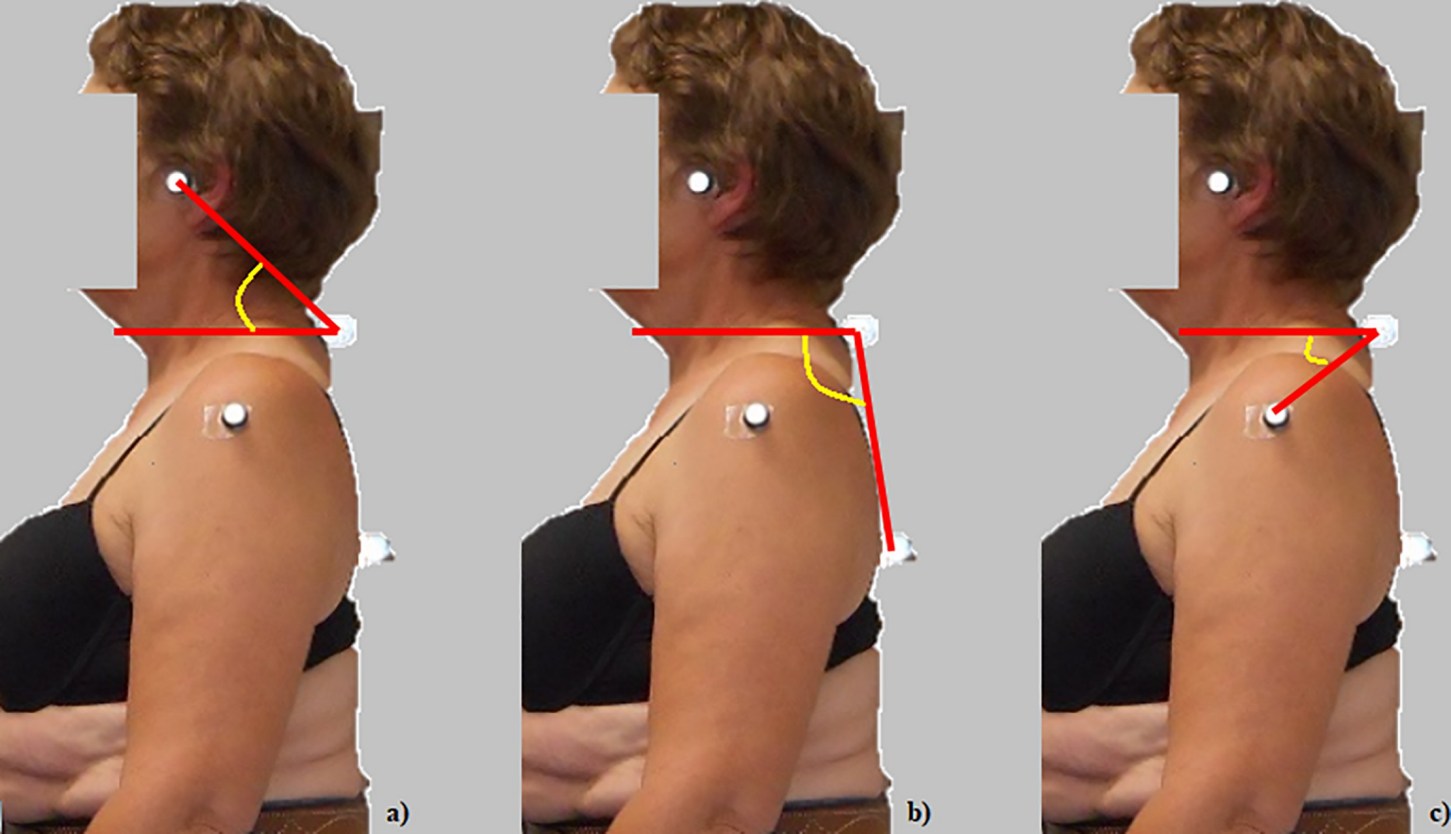
Posture angles—***a*:** Head posture (craniocervical angle); ***b*:** upper back posture (cervicothoracic angle); ***c***: shoulder posture (shoulder protraction angle).

Posture was also assessed using digital photography with the camera set-up as outlined above. Three angles were calculated from lateral photographs taken of participants in standing to indicate head [31], upper back [32] and shoulder posture [33] (Fig 2A–2C). Photo-reflective adhesive markers were fitted to the anatomical landmarks of interest (C7 and T7 spinous processes; tragus of the ear; and lateral mid-point of the humeral head) before participants were instructed to stand comfortably for a single left-sided photograph. Images were digitised using ImageJ software (National Institutes of Health, Bethesda, MD). Head posture (craniovertebral angle) was determined from the angle formed between a line drawn from the tragus of the ear to the seventh cervical vertebrae subtended to the horizontal (Fig 2A). Upper back posture (cervicothoracic angle) was determined from the angle between a horizontal line and a line between the seventh cervical and seventh thoracic vertebrae. (Fig 2B). Shoulder posture (shoulder protraction angle) was determined from the angle formed between a horizontal line and a line between the mid-point of the shoulder and C7 (Fig 2C).

### Statistical analysis

A priori sample size calculation determined that a sample of at least 100 would be sufficient to detect change in odds for UBP of 0.9 or larger with a power of 80% and a confidence level of 95% [34]. Data were analyzed using SPSS version 24 (IBM; Chicago, IL) and STATA version 15.1 (StatCorp LP, College station, TX). Descriptive statistics were calculated for self-report and physical characteristics.

### Between-group differences

The differences between groups were examined using independent-samples t-tests or Chi-square test as appropriate.

### UBP risk

Each physical characteristic was then evaluated separately in a logistic regression model adjusted for age. Odds ratios were standardised by calculating them for a one standard deviation (SD) change in each variable of interest. Those variables significantly associated with UBP after adjustment for age, were entered into a multivariable model. Age was manually entered in step one and physical characteristic variables were entered forwards stepwise into the model in step 2 with probability of removal at p<0.05. The total explained variance in UBP (Yes/No) explained by the model (Nagelkerke R-squared, R^2^) and physical characteristics that were statistically significant were identified. The model was confirmed using the backward stepwise method. The assumptions and standardised residual values of each model were checked and sensitivity analysis was performed where outliers (standardised residuals >2.5) were identified to be certain and confident in the model output.

### UBP severity

Censored regression (Tobit model) analysis was used to assess univariate linear relationships between each continuous physical characteristic and UBP severity (UBP NRS). Those characteristics that were significantly associated with UBP severity after adjustment for age were entered into a multivariable model. A hierarchical Tobit multivariable regression model adjusted for age in step one was built using physical characteristics entered as z-scores. Those characteristics with a statistically significant contribution (p<0.05) to the final model were identified.

## Results

A total of 119 postmenopausal women with mean (SD) age 61.4 (7.0) years; height 161.4 (6.2) cm; weight 75.2 (15.2) kg; and BMI 28.9 (5.5) kg/m^2^ participated in this study. Sixty-one participants (51%) had UBP. Of these, 57 (93%) reported chronic UBP (>3 month duration). The severity of UBP reported by women with UBP ranged from 1 to 10 on the NRS with a mean (SD) of 4.56 (2.11).

### Between group differences

Participants with UBP were younger, had larger breasts, greater ptosis and less upper back extensor muscle endurance (Table 1).

**Table 1:** Logistic regression analysis. Physical characteristics and the association with UBP (yes/no).

Physical Characteristic		Descriptive Data				Multivariable Analysis			
		Whole sample (n = 119)	Nil UBP group (n = 58)	UBP group (n = 61)	Between group comparisons	Step 1: Adjustment for age		Final Model^d^ Step 1: age Step 2: predictor variables (n = 119)	
		Mean (SD)	Mean (SD)	Mean (SD)	Mean Difference (95% CI)	Odds ratio (95%CI)	p-value	Odds ratio (95%CI)	p-value
Age (yrs)		61.4 (7.0)	63.8 (6.6)	59.1 (6.6)	**-4.7 (-7.1 to 2.3)**	**0.90 (0.85–0.96)**	**<0.001**	**0.35 (0.21–0.58)**	**<0.001**
Height (cm)		161.4 (6.2)	162.4 (5.7)	160.5 (6.5)	-1.9 (-4.1 to 0.32)	**0.57 (0.37–0.87)**^d^	**0.010**	**0.50 (0.31–0.79)**	**0.003**
Weight (kg)		75.2 (15.2)	73.9 (15.0)	76.1 (15.5)	2.6 (-2.9 to 8.2)	1.22 (0.83–1.79)	0.320		
BMI (kg/m^2^)		28.9 (5.5)	28.0 (5.3)	29.7 (5.6)	1.7 (-0.3 to 3.7)	**1.55 (1.02–2.35)**	**0.039**		0.289^e^
BMD (g/cm^2^)		0.9 (0.1)	0.9 (0.1)^a^	0.9 (0.1)^a^	0.2 (-0.02 to 0.1)	1.06 (0.72–1.58)	0.760		
Lean mass (kg)		40.6 (5.7)	56.2 (6.8)	54.3 (6.1)^a^	-0.3 (-2.4 to 1.8)	0.90 (0.61–1.33)	0.573		
Fat mass (kg)		31.7 (10.3)	42.0 (7.1)	44.0 (6.5)^a^	2.0 (-1.7 to 5.8)	1.31 (0.88–1.95)	0.168		
Breast size (BSS)		7.0 (3.2)	6.4 (2.8)	7.6 (3.4)	**1.2 (0.9 to 2.3)**	**1.54 (1.01–2.34)**	**0.043**		0.113^e^
Ptosis (cm)		25.6 (4.0)	24.8 (3.5)	26.4 (4.2)	**1.6 (0.23 to 0.2)**	**1.67 (1.08–2.60)**^d^	**0.023**		0.078^e^
Breast splay (cm)		24.3 (3.9)	23.7 (3.6)	24.8 (4.1)	1.1 (-0.3 to 2.4)	1.35 (0.91–2.01)	0.143		
Bra Fit	Pass	-	24 (41.4)^c^	19 (31.1)^c^	**-**	0.65 (0.29–1.43)	0.283		
	Fail		34 (58.6)^c^	42 (68.1)^c^					
Upper back extensor muscle endurance (s)		99.1 (72.6)	117.3 (85.3)	81.8 (53.2)	**-35.5 (-61.5 to -9.8)**	**0.52 (0.33–0.82)**	**0.005**	**0.46 (0.28–0.75)**	**0.002**
Back mobility (°)		9.3 (4.9)	9.8 (5.2)	8.8 (4.5)	-1.0 (-2.7 to 0.8)	0.85 (0.58–1.25)	0.409		
Thoracic Kyphosis centroid angle^b^ (°)		42.2 (10.9)	41.8 (10.4)^a^	42.7 (11.5)^a^	0.9 (-3.1 to 4.9)	1.29 (0.86–1.94)	0.215		
Head posture (°)		38.8 (6.6)	39.1 (6.9)	38.6 (6.4)	-0.5 (-1.9 to 2.9)	0.78 (0.52–1.17)	0.223		
Upper back posture (°)		104.7 (5.6)	104.4 (5.7)	103.7 (12.4)	0.7 (-1.4 to 2.7)	1.31 (0.87–1.96)	0.197		
Shoulder posture (°)		30.3 (9.1)	29.9 (9.5)	30.7 (8.8)	0.8 (-2.5 to 4.1)	1.17 (0.80–1.73)	0.422		
Thoracic Osteoarthritis	Nil/mild	-	36 (62.1)^c^	33 (51.4)^b^	-	0.56 (0.25–1.24)	0.152		
	Moderate/severe		21 (36.2)^c^	27 (44.3)^c^					
Thoracic Fracture	Nil Fracture	-	53 (91.4)^c^	47 (77.0)^c^	-	**0.12 (0.23–0.58)**^d^	**0.009**		0.151^e^
	Fracture		4 (6.9)^c^	13 (21.3)^c^					

^a^ One missing value

^b^ Two missing values

^c^ Categorical variables values presented as n (%)

^d^<3 outliers remained in analysis

^e^ Variable removed in final model (p>0.05)

**Abbreviations:** UBP—Upper back pain; BMI—Body mass index; BMD—Bone mineral density; BSS—Breast size score; 95%CI: 95% confidence interval; cm–centimetres; kg–kilograms; kg/cm^2 –^kilograms per centimeter-squared; s–seconds

### UBP risk

In univariate models adjusted for age, a higher BMI, larger breasts and greater ptosis were physical characteristics associated with a significant *increase* in odds for UBP (Table 1). Conversely, greater height, better upper back extensor muscle endurance, and the absence of a thoracic vertebral fracture were associated with a significant *decrease* in odds for UBP (Table 1). In the multivariable model, after adjusting for age, each SD (72.6s) increase in upper back extensor muscle endurance was significantly associated (p = 0.002) with 54% decrease in the odds of UBP and each SD (6.2cm) increase in height was significantly associated (p = 0.003) with a 50% decrease in the odds of UBP (Table 1). The age adjusted multivariable logistic regression model significantly Χ^2^ (3) = 30.94, p<0.001, explained 31% (R^2^) of the variance in UBP. The sensitivity analysis indicated that there was no change to this model by excluding two standardised residuals with a value of >2.5.

### UBP severity

After adjustment for age there were six physical characteristics significantly associated with UBP severity (Table 2). Being taller, leaner and having smaller breasts, less ptosis, greater upper back extensor muscle endurance and better back mobility were significantly associated with lower severities of UBP. The multivariable model, adjusted for age and containing these physical characteristic variables identified lean mass and upper back extensor muscle endurance as the physical characteristics that independently explained differences in UBP severity. With a one standard deviation increase in age (7.0-years), lean mass (5.7kg) or upper back extensor muscle endurance (72.6), there was a significant reduction in UBP NRS score by 0.95, 0.73 and 0.91-points respectively.

**Table 2:** Censored (Tobit) regression analysis: Physical characteristics and the association with UBP severity (NRS).

	Tobit model regression (n = 119)								
Physical characteristic	Univariate Analysis			Step 1: Adjustment for age			Multivariable analysis Step 1: Age Step 2: Predictor variables		
	Tobit regression coefficient (95% CI)	Std. error	p-value	Tobit regression coefficient (95% CI)	Std. error	p-value	Tobit regression coefficient (95%CI)	Std. error	p-value
**Age (yrs)**	**-1.04 (-1.04 to -0.62**	**0.21**	**<0.001**				**-0.95** (**-1.38 to -0.52)**	**0.22**	**<0.001**
**Height (cm)**	**-0.62 (-1.10 to-0.14)**	**0.24**	**0.013**	**-0.84 (-1.29 to -0.38)**	**0.23**	**<0.001**			0.112^c^
Weight (kg)	-0.04 (-0.53 to 0.46)	0.25	0.884	-0.03 (-0.50 to 0.44)	0.24	0.892			
BMI (kg/m^2^)	0.20 (-0.30 to 0.70)	0.25	0.424	0.29 (-0.18 to 0.76)	0.24	0.229			
BMD (g/cm^2^)^a^	0.11 (-0.39 to 0.61)	0.25	0.660	-0.08 (-0.57 to 0.41)	0.25	0.748			
**Lean mass (kg)**^**b**^	**-0.51 (-1.00 to -0.02)**	**0.25**	**0.040**	**-0.58 (-1.04 to -0.12)**	**0.23**	**0.015**	**-0.73 (-1.17 to -0.29)**	**0.22**	**0.001**
Fat mass (kg)^**b**^	0.07 (-0.43 to 0.57)	0.25	0.780	0.13 (-0.34 to 0.60)	0.24	0.592			
**Breast size (BSS)**	**0.50 (0.01 to 0.99)**	**0.25**	**0.044**	**0.47 (0.00 to 0.93)**	**0.23**	**0.049**			0.945^c^
**Ptosis (cm)**	**0.61 (0.12 to 1.09)**	**0.24**	**0.014**	**0.63 (0.17 to 1.08)**	**0.23**	**0.008**			0.063^c^
Breast splay (cm)	0.40 (-0.09 to 0.89)	0.25	0.110	0.37 (-0.10 to 0.83)	0.24	0.123			
**Upper back extensor muscle endurance (s)**	**-0.76 (-1.23 to -0.28)**	**0.24**	**0.002**	**-0.81 (-1.26 to -0.36)**	**0.23**	**0.001**	**-0.91 (-1.35 to -0.48)**	**0.22**	**<0.001**
**Back mobility (°)**	**-0.57 (-1.06 to -0.09)**	**0.25**	**0.021**	**-0.50 (-0.97 to -0.04)**	**0.23**	**0.035**			0.592^c^
Thoracic Kyphosis centroid angle^**b**^ (°)	0.24 (-0.26 to 0.74)	0.25	0.343	0.43 (-0.05 to 0.91)	0.24	0.082			
Head posture (°)	-0.05 (-0.55 to 0.44)	0.25	0.832	-0.23 (-0.71 to 0.25)	0.24	0.349			
Upper back posture (°)	0.17 (-0.33 to 0.66)	0.25	0.510	0.32 (-0.16 to 0.79)	0.24	0.190			
Shoulder posture (°)	0.17 (-0.32 to 0.67)	0.25	0.490	0.23 (-0.24 to 0.71)	0.24	0.326			

^a^ Two missing values

^b^ One missing value

^c^ Variable removed in final model (p>0.05).

**Abbreviations:** BMI—Body mass index; BMD—Bone mineral density BSS—Breast size score; 95%CI—95% confidence interval; Std error—standard error; cm—centimeter; kg—kilograms; kg/m^2^—kilograms per metre-squared; g/cm^2^—grams per centimeter-squared; s—seconds.

## Discussion

The purpose of this research was to better understand UBP in postmenopausal women by identifying associated factors. Using an exploratory approach that, for the first time, examined several physical characteristics simultaneously in a single study, we have identified that independent of age, upper back extensor muscle endurance is the only physical characteristics that contributes to explaining variance in both the risk and severity of UBP in postmenopausal women.

Participants with UBP in this study differed overall from those without UBP on the basis of very few physical characteristics before adjusting for age. It was an unexpected finding that the odds for UBP and ratings of its severity were lower in the older participants. We are not the first to identify an inverse relationship between age and presence or severity of back pain [18, 35, 36]. The reasons for this have not been prospectively explored but could relate to a change in pain perception with age [37] or could reflect trends showing that increased musculoskeletal pain is not an inevitable consequence of ageing [38].

Risk factors for UBP have not been previously examined as a collective group in postmenopausal women. Concurrent musculoskeletal symptoms and difficulty performing activities of daily living have been previously reported as being associated with UBP in adults [4]. It is unknown whether these are, age or gender specific. In studying a broad range of physical characteristics, the current study suggests the importance that upper back extensor muscle endurance may have, over and above other physical characteristics, in the aetiology of UBP in postmenopausal women. Physical characteristics such as poor posture, more severe osteoarthritis, prevalent vertebral fractures, larger thoracic kyphosis, lower back mobility and higher BMI, which anecdotally are considered common physical features of people with UBP, have been shown to be less important amongst the participants of our study. Since this is the first study to collectively assess this many different physical characteristics and the first to examine their relative importance to UBP, the results are novel and require further substantiation. It is possible, for example, that by excluding volunteers with known thoracic spine pathology that it made some physical characteristics, such as prevalent vertebral fractures and thoracic hyperkyphosis, less detectable. This may also explain the low average UBP severity scores (NRS of 4.5-points) recorded across the sample. The ‘mild’[39] severity of these average scores may also have precluded us from identifying relationships between some physical characteristics and UBP severity.

Back extensor muscle endurance has not previously been investigated as a risk factor for UBP although it has been reported, as a characteristic that increases the odds for low back pain [12, 40] and chronic neck pain [13]. By showing that UBP becomes less likely with increasing levels of upper back extensor muscle endurance, our results suggest that this could be a potent targetable characteristic of postmenopausal women that may offset their risk of UBP.

Although the mechanism between upper back extensor muscle endurance and UBP remains unclear there is evidence that back muscle function can affect thoracic spinal compressive loading and that this may contribute to the development of UBP [41]. Changes to spinal loading as a result of poor back muscle endurance and the implications for spinal pain is a longstanding concept in low back pain research [42]. Since upper back extensor muscles have a primarily postural function [26] it is plausible that deficits in endurance could affect spinal loading over time which may in turn become symptomatic. Training back muscle function as a way of mitigating the compressive loading of the thoracic spine improves thoracic postures [43–47] and reduces the risk of vertebral fractures [48, 49]. The range of simple back extensor exercises, that are previously-described to have such benefits are well-tolerated by postmenopausal women with and without pathology affecting the thoracic spine [44–49]. An important direction for future research is to evaluate if the therapeutic benefits of such exercises extend to improving upper back extensor muscle endurance and beyond this to reducing the risk and severity of UBP in postmenopausal women.

It was of interest that both breast size and breast ptosis were physical characteristics associated with both the risk and severity of UBP in univariate models which helps to corroborate that UBP may have unique risk factors such as these. This is of particular relevance to older women who commonly experience an increase in breast size [16, 50, 51]. The elevated risk of UBP with increasing breast size verifies previous reports in postmenopausal [15] and younger women [14, 18]. This study has shown further that, in relation to other physical characteristics, the importance of breast size to the risk and severity of UBP may not be as substantial as previously suggested. Breast size has been descriptively linked to UBP via putative biomechanical mechanisms in studies of women with different breast sizes where other physical characteristics such as thoracic kyphosis and posture have been implicated [14, 52, 53]. The current study has been unable to confirm that thoracic kyphosis or posture (head, upper back or shoulder posture) are physical characteristics that are related to UBP risk or its severity. Whilst larger breast sizes could plausibly contribute to UBP, the role and relevance of thoracic kyphosis or posture as mediators of the relationship between breast size and UBP needs more substantiation. Further prospective investigation of the relationships between breast size and physical characteristics, alongside UBP, is needed to confirm a causative link and mechanism. Examining the potentially important role of upper back extensor muscle endurance as a factor with clear links to the risk and severity of UBP could be worthwhile.

Another possible unique risk factor for UBP related to breast size is breast support from a correctly fitted bra. This has been speculated as a source of UBP in women with large breasts [14]. Women with large breasts commonly wear ill-fitting bras [24, 54, 55] and are considered more likely to experience UBP as a result of this [56]. The value of a correctly fitted bra has been previously demonstrated to reduce breast pain [57] and improve comfort with physical activity amongst women with large breasts [58, 59]. However, correct bra fit has not been previously examined as a factor related to UBP. Since there was no significant association between bra fit and UBP, our findings do not support the concept that a correctly-fitted bra may “protect” against UBP in postmenopausal women [56]. Whether this is dependent on the size of a woman’s breasts or the specific characteristics of her bra, requires further exploration.

In this study, upper back extensor muscle endurance, age and height were physical characteristics that collectively explained less than half (31%) of the variance in UBP risk, suggesting there are other characteristics, that have not been examined here, that may account for the remaining 69% of the variance. Furthermore, the characteristics of age, upper back extensor muscle endurance and lean body mass contributed only very small amounts to changes in UBP severity and of note, only one of these is reasonably easy to change for postmenopausal women. These outcomes suggest that further work is needed to identify factors associated with the risk and severity of UBP in postmenopausal women. This could include an examination of psychosocial characteristics, which have received little attention in the study of UBP in the past but could, nevertheless, be of importance [4].

The findings of this study should be considered in the context of its limitations. The study considered what physical characteristics may be risk factors for UBP. Causal relationships cannot be assumed, due to the cross- sectional design of our study. Upper back pain quantified using the NRS revealed relatively low levels of pain severity across participants in our sample. This may have concealed some of the relationships that we could identify between physical characteristics and UBP risk and severity. Targeting a sample with higher pain scores may overcome this limitation in the future.

In summary, this study concludes that, irrespective of age, postmenopausal women may be less likely to suffer from UBP and severe pain if they maintain good upper back extensor muscle endurance. There is some evidence that breast characteristics, including size and ptosis, are characteristics that are relevant to UBP in postmenopausal women but as risk factors these are less conspicuous.

## Supporting information

S1 Appendix Breast size score conversion chart. (DOCX)
