# Supplementary material for: Upper back pain in postmenopausal women and associated physical characteristics
Source: PLoS One. 2019 Jul 31;14(7):e0220452. doi: 10.1371/journal.pone.0220452 (PMC6668906; doi:10.1371/journal.pone.0220452)
Supplement: S1 Appendix — (DOCX) [file pone.0220452.s001.docx]

**S1 Appendix** Breast Size Score (BSS) Conversion Chart^a^

|  |  | **Band size** (under-bust circumference, cm) | | | | | | | | | |
| --- | --- | --- | --- | --- | --- | --- | --- | --- | --- | --- | --- |
|  | **Cup size** | **8**  (63-67) | **10**  (68-72) | **12**  (73-77) | **14**  (78-82) | **16**  (88-87) | **18**  (88-92) | **20**  (93-97) | **22**  (98-102) | **24**  (103-107) | **26**  (108-112) |
| **Breast size score (**over-bust circumference, cm**)** | **AA** | **0**  (75-77) | **1**  (80-82) | **2**  (85-87) | **3**  (90-92) | **4**  (95-97) | **5**  (100-102) | **6**  (105-107) | **7**  (110-112) | **8**  (115-117) | **9**  (120-122) |
|  | **A** | **1**  (77-79) | **2**  (82-84) | **3**  (87-89) | **4**  (92-94) | **5**  (97-99) | **6**  (102-104) | **7**  (107-109) | **8**  (112-114) | **9**  (117-119) | **10**  (122-124) |
|  | **B** | **2**  (79-81) | **3**  (84-86) | **4**  (89-91) | **5**  (94-96) | **6**  (99-101) | **7**  (104-106) | **8**  (109-111) | **9**  (114-116) | **10**  (119-121) | **11**  (124-126) |
|  | **C** | **3**  (81-83) | **4**  (86-88) | **5**  (91-93) | **6**  (96-98) | **7**  (101-103) | **8**  (106-108) | **9**  (111-113) | **10**  (116-118) | **11**  (121-123) | **12**  (126-128) |
|  | **D** | **4**  (83-85) | **5**  (88-90) | **6**  (93-95) | **7**  (98-100) | **8**  (103-105) | **9**  (108-110) | **10**  (113-115) | **11**  (118-120) | **12**  (123-125) | **13**  (128-130) |
|  | **DD** | **5**  (85-87) | **6**  (90-92) | **7**  (95-97) | **8**  (100-102) | **9**  (105-107) | **10**  (110-112) | **11**  (115-117) | **12**  (120-122) | **13**  (125-127) | **14**  (130-132) |
|  | **E** | **6**  (87-89) | **7**  (92-94) | **8**  (97-99) | **9**  (102-104) | **10**  (107-109) | **11**  (112-114) | **12**  (117-119) | **13**  (122-124) | **14**  (127-129) | **15**  (132-134) |
|  | **F** | **7**  (89-91) | **8**  (94-96) | **9**  (99-101) | **10**  (104-106) | **11**  (109-111) | **12**  (114-116) | **13**  (119-121) | **14**  (124-126) | **15**  (129-131) | **16**  (134-136) |
|  | **G** | **8**  (91-93) | **9**  (96-98) | **10**  (101-103) | **11**  (106-108) | **12**  (111-113) | **13**  (116-11)8 | **14**  (121-123) | **15**  (126-128) | **16**  (131-133) | **17**  (136-138) |
|  | **H** | **9**  (93-95) | **10**  (98-100) | **11**  (103-105) | **12**  (108-110) | **13**  (113-115 | **14**  **(**118-120) | **15**  (123-125) | **16**  (128-130) | **17**  (133-135) | **18**  (138-140) |

^a^ To determine a breast size score first identify the correct bra size (band and cup size). Establish the correct band size by measuring around the body, directly below the bust (under-bust circumference) and the correct cup size by measuring across the fullest part of the breasts whilst wearing a bra (over-bust circumference) (Berlei, 2018). Use the top row of the table to select the band size, this increases from left to right. Then use the first column of the table to select the cup size, this increases from top to bottom. Track down and across to find the table cell where these two selections intersect. Breast size score is shown in bolded text.
